# Altered mTOR and Beclin-1 mediated autophagic activation during right ventricular remodeling in monocrotaline-induced pulmonary hypertension

**DOI:** 10.1186/s12931-017-0536-7

**Published:** 2017-03-24

**Authors:** Yan Deng, Weifeng Wu, Shenglan Guo, Yuming Chen, Chang Liu, Xingcui Gao, Bin Wei

**Affiliations:** 1 Department of Ultrasound, First Affiliated Hospital of Guangxi Medical University, Nanning, People’s Republic of China; 2 Department of Cardiology, First Affiliated Hospital of Guangxi Medical University, 6 Shuangyong Road, Nanning, 530021 People’s Republic of China

**Keywords:** Autophagy, Right ventricular, Cardiac remodeling, Pulmonary hypertension, Monocrotaline

## Abstract

**Background:**

Right ventricular structure and function is a major predictor of outcomes in pulmonary hypertension (PH), yet the underlying mechanisms remain poorly understood. Growing evidence suggests the importance of autophagy in cardiac remodeling; however, its dynamics in the process of right ventricle(RV) remodeling in PH has not been fully explored. We sought to study the time course of cardiomyocyte autophagy in the RV in PH and determine whether mammalian target of rapamycin (mTOR) and Beclin-1 hypoxia-related pro-autophagic pathways are underlying mechanisms.

**Methods:**

Rats were studied at 2, 4, and 6 weeks after subcutaneous injection of 60 mg/kg monocrotaline (MCT) (MCT-2 W, 4 W, 6 W) or vehicle (CON-2 W, 4 W, 6 W). Cardiac hemodynamics and RV function were assessed in rats. Autophagy structures and markers were assessed using transmission electron microscope, RT-*q*PCR, immunohistochemistry staining, and western blot analyses. Western blot was also used to quantify the expression of mTOR and Beclin-1 mediated pro-autophagy signalings in the RV.

**Results:**

Two weeks after MCT injection, pulmonary artery systolic pressure increased and mild RV hypertrophy without RV dilation was observed. RV enlargement presented at 4 weeks with moderately decreased function, whereas typical characteristics of RV decompensation and failure occurred at 6 weeks thus demonstrating the progression of RV remodeling in the MCT model. A higher LC3 (microtubule- associated protein light chain 3) II/I ratio, upregulated LC3 mRNA and protein levels, as well as accumulation of autophagosomes in RV of MCT rats indicated autophagy induction. Autophagy activation was coincident with increased pulmonary artery systolic pressure. Pro-autophagy signaling pathways were activated in a RV remodeling stage-dependent manner since phospho-AMPK (adenosine monophosphate-activated protein kinase)-α were primarily upregulated and phospho-mTOR suppressed in the RV at 2 and 4 weeks post-MCT injection, whearas, BNIP3 (Bcl2-interacting protein 3) and beclin-1 expression were relatively low during these stages, they were significantly upregulated after 6 weeks in this model.

**Conclusions:**

Our findings provide evidence of sustained activation of autophagy in RV remodeling of MCT induced PH model, while pro-autophagic signaling pathways varied depending on the phase.

## Background

Pulmonary hypertension (PH) is a devastating and fatal disease characterized by pulmonary vascular remodeling and vasoconstriction, resulting in increased pulmonary vascular resistance and subsequent right ventricular (RV) hypertrophy and heart failure [1–3]. It has been established that the RV function is the most important prognostic factor for both morbidity and mortality ﻿of patients with PH [1–3]. Current interventions aimed at improving RV function are suboptimal, [4, 5] which may be partly due to the limited insights on the mechanisms driving the progression of the RV from hypertrophy to failure [2, 6, 7]. As a result, preserving RV function is emerging as a critical research priority in the cardiopulmonary research field [3].

Autophagy, a highly conserved lysosomal-dependent process for the bulk degradation and recycling of long-lived proteins and organelles, has recently emerged in playing a pivotal role in cardiac remodeling [8]. Autophagy serves as a major mechanism for cellular survival in cardiomyocytes [8, 9]. Paradoxically, excessive autophagy may result in cardiomyocyte death and contribute to cardiac dysfunction [8, 10, 11]. Microtubule associated protein light chain 3 (LC3) is a crucial marker of autophagy [10]. LC3 initially yields a cytosolic form LC3-I, which is converted to LC3-II during the formation of autophagosomes. LC3-II is recruited to the expanding autophagosomal membranes and associates specifically with autophagosomes [12]. Thus, LC3 and the ratio of LC3-II/LC3-I are considered markers of autophagy. Autophagosomes can be observed using a transmission electron microscope and are considered “gold standards” in terms of morphological/ultrastructural evidence of autophagy activation, [13] while upregulation of p62 poses as a marker of autophagosomal clearance [14, 15].

Presently, the disease-modifying effects of autophagy in the progression of RV remodeling and the underlying mechanisms remain poorly understood. Qipshidze et al. were the first to demonstrate autophagy changes in the RV of a pulmonary artery banding mouse model [16]. However, this model is associated with longer periods of compensation, which clinically more closely mimics RV remodeling in pulmonary stenosis [2]. A widely accepted experimental model of PH is generated by injection of monocrotaline (MCT), a pyrrolizidine alkaloid toxin that selectively affects the pulmonary vasculature [17]. The MCT induces PH, which mediates a gradual rise in pressure load on the RV. This can induce compensatory RV myocardial hypertrophy after the second week and chamber expansion after 3–4 weeks, and result in marked RV dilatation and global hypokinesis at 5–6 weeks [2, 7]. Thus, the MCT model is characterized by RV failure similar to humans, and is characterized by decreased activity, ascites, overall weight loss, and increased mortality [2, 3, 7]. Although the impact of autophagy has been extensively studied in the pulmonary vasculature in the MCT model, [18, 19] there is a paucity of data concerning the impact of autophagy in the RV in the model [20].

The exact mechanisms underlying autophagic activation remain to be delineated precisely, however there is emerging evidence for a critical role of autophagy signaling in adaptive and maladaptive responses [21]. There are two crucial pathways involved in the regulation of autophagy during ischemia/hypoxia. One is the adenosine monophosphate-activated protein kinase (AMPK)/mammalian target of rapamycin (mTOR) and the other is hypoxia inducible factor 1α (HIF-1α)/B-cell lymphoma 2 (Bcl2)/adenovirus E1B 19 kDa protein-interacting protein 3 (BNIP3)/Beclin-1 pathway [22, 23]. RV capillary rarefaction, decreased coronary artery perfusion, reduction in RV myoglobin protein, and an enlargement of RV cardiomyocytes leading to RV myocardial ischemia/hypoxia are structural derangements contributing to the progression of RV failure in PH [3, 24–27]. Thus, we hypothesized that RV autophagy would be activated through one or both of these signaling pathways. Therefore, we aimed to establish the dynamics and potential mechanisms of autophagy in the RV in vivo during RV remodeling in the MCT rat model. These findings will help to provide new mechanistic insights into pathways that drive autophagy-mediated RV remodeling in PH.

## Methods

### Animal model

Animal protocols were approved by the Ethics Committee of Animal Experiments at Guangxi Medical University and conformed to the Guide for the Care and Use of Laboratory Animals published by the National Institutes of Health. Pathogen-free inbred male Sprague Dawley rats were purchased from the Guangdong Laboratory Animal Center at the Chinese Academy of Sciences (Certificate No. SCXK [Yue] 2013–0002) and bred in a pathogen-free mouse room at the Experimental Animal Center (Guangxi Medical University, China). Rats were housed under controlled temperatures (22 °C) in a humidity-controlled room using a 12-h light:12-h dark cycle. All rats received standard rat chow and water *ad libitum*. The rats (8-weeks-old, weight 250 to 280 g, *n* = 60) were randomly assigned to either the MCT (*n* = 36) or control (*n* = 24) group. The MCT group received a single 60 mg/kg subcutaneous injection of MCT (Sigma-Aldrich, St. Louis, MO, USA), while the control group received an equivalent volume of saline. Within each group, rats were further subdivided according to injection durations (2 weeks, 4 weeks, 6 weeks). These subgroups were termed MCT-2 W (*n* = 12), MCT-4 W (*n* = 12), and MCT-6 W (*n* = 12) as well as CON-2 W (*n* = 8), CON-4 W (*n* = 8), and CON-6 W (*n* = 8).

### Transthoracic echocardiography

Echocardiography was performed at 2, 4, and 6 weeks. The rats were anesthetized by intraperitoneal administration of 3% pentobarbital sodium (50 mg/kg) and subjected to transthoracic echocardiography using a HP Sonos 7500 system (Philips Medical Systems, Andover, MA, USA) equipped with a 12.0 MHz phase array transducer (S12-4 scanner). Echocardiography was performed according to the recommendations of the American College of Echocardiography [28]. RV wall thickness (RVWT), right ventricular end-diastolic diameter (RVEDD), RV end-systolic and end-diastolic areas (RVEDA, RVESA), as well as Volumes (RVEDV, RVESV) were assessed in the apical four-chamber view. RV fractional area change (RV FAC) was calculated using the formula: RV FAC (%) = (RVEDA-RVESA)/RVEDA × 100%. RV ejection fraction (RVEF) was calculated using the formula: RVEF (%) = (RVEDV-RVESV)/RVEDV × 100%. Tricuspid annular plane systolic excursion (TAPSE) was obtained in M-mode as the displacement of the lateral tricuspid annulus. Pulmonary artery acceleration time (PAAT) assessed by pulse Doppler was acquired as reported previously [29, 30]. All evaluations were performed by an observer who was blinded to the groups and each parameter was averaged over six cardiac cycles.

### Invasive right heart hemodynamic measurements

After echocardiography, the rats were anesthetized as described above and placed in a supine position on a heated pad. Using a closed chest model, an experienced anesthesiologist isolated and cannulated the right external jugular vein using a 2 F high fidelity microtip pressure catheter under an Olympus CH30 microscope (Olympus, Tokyo, Japan). The catheter was then inserted into the superior vena cava (SVC), right atrium (RA), right ventricle, and pulmonary artery as previously described [31, 32]. Catheter position was determined in accordance with changes in the pressure waveform. Pressure waveforms were low in the SVC and slightly increased in pulsatility in the RA. When the catheter was inserted into the RV, a waveform occurred as evidenced by a large step during systole and a pressure drop to near zero during diastole. Pulmonary artery systolic pressure (PASP) was similar to the max RV pressure; however, the diastolic pressure was elevated. Pulmonary artery waveforms were recorded after a 10 min equilibration period with the ALCB10 Heart Function Analysis System (Shanghai Alcott Biotech Co. Ltd, Shanghai, China). Data were averaged from six consecutive cardiac cycles.

### Histopathological analysis

After invasive hemodynamic analysis, RV, left ventricular free wall (LV) and interventricular septal (IVS) tissues were carefully separated, harvested, and weighed from anesthetized rats. Right ventricular hypertrophy was expressed as right ventricular weight (RV free wall) over body weight (RV/BW) and left ventricular plus interventricular septum weight [RV/(LV + IVS)] [2, 33, 34]. RV and LV were flushed with physiological saline solution and fixed with 10% formalin, and then embedded in paraffin. Tissues were subjected to sectioning (5 μm thickness) as well as hematoxylin and eosin (H&E) stain. Cardiac fibrosis was analyzed by staining RV tissue sections with Masson’s trichrome stain, and collagen volume fraction (CVF) was assessed as previously described [35]. Cardiomyocyte cross-sectional area was assessed by staining RV tissues with wheat germ agglutinin conjugated to Alexa Fluor® 488 dye (WGA, Invitrogen, Carlsbad, CA, USA) and counterstained for nuclei with 4, 6-diamidino-2-phenylindole (DAPI, Sigma-Aldrich, St. Louis, MO, USA). Images were captured using an Olympus BP80 microscope and software (Olympus, Tokyo, Japan). Using Image-Pro Plus v6.0 software (Media Cybernetics, Rockville, MD, USA), 15 slices of RV tissue were evaluated for each animal.

### Quantitative real time-polymerase chain reaction

The mRNA expression of autophagy marker LC3 was quantified using real-time quantitative polymerase chain reaction (RT-*q*PCR). Total RNA was isolated from rat RV and LV free wall tissues using TRIzol (Invitrogen, Carlsbad, CA, USA), and then subjected to reverse transcription into cDNA using a Reverse Transcription Kit (Takara, Dalian, China). The purity of RNA (260/280 nm ratio) was determined spectrophotometrically using the NanoDrop 2000 spectrophotometer (Thermo Fisher Scientific Inc., Rockford, IL, USA). Two-step qRT-PCR was used to quantify relative LC3 mRNA by the ABI PRISM® 7500 qPCR System (Applied Biosystems, Foster City, CA, USA) using SYBR Green. The relative LC3 mRNA expression was normalized to the level of glyceraldehyde-3 phosphate dehydrogenase (GAPDH) using ΔΔC_T_ method. Each reaction was carried out in triplicate. Using Primer Premier v5.0 software (PREMIER Biosoft International, Palo Alto, CA, USA), primers for LC3 and GAPDH were designed as follows:

- rat LC3 (NCBI accession number: NM_199500.2): 5′-CGAGAGCGAGAGAGATGAAGACGG-3′ (sense) and 5′-GGTAACGTCCCTTTTTGCCTTGGTA-3′ (antisense);
- rat GAPDH (NCBI accession number: NM_017008): 5′-TCCATGACAACTTTGGCATCGTGG-3′ (sense) and 5′-GTTGCTGTTGAAGTCACAGGAGAC-3′ (antisense).

### Immunohistochemistry

Deparaffinized myocardial tissue sections were subjected to 3% hydrogen peroxide for 15 min. For heat-induced epitope retrieval, the sections were placed in a 0.01 mol/L (pH 6.0) citrate buffer and heated at 120 °C for 10 min. Nonspecific binding was blocked by pre-incubation with 5% goat serum in phosphate-buffered saline for 20 min at room temperature. The sections were incubated with a rabbit anti-LC3 antibody (diluted 1:3200, Cell Signaling, Danvers, MA, USA) in blocking buffer for overnight at 4 °C. Phosphate-buffered saline was used to wash sections. Sections and were then incubated with streptavidin-biotin complex at room temperature for 20 min. Following extensive phosphate-buffered saline washes, sections were subjected to 2% 3,3′-diaminobenzidine in 50 mmol/L Tris buffer (pH 7.6) containing 0.3% hydrogen peroxide for 5–10 min to develop the color reaction. Sections were imaged (15 sections per rat) for LC3 positivity with an Olympus BX51 microscope/software (Olympus, Tokyo, Japan) and Image-Pro Plus v6.0 software (Media Cybernetics, Rockville, MD, USA). Two pathology experts blindly scored and analyzed five random fields from each section and plotted data using mean integrated optical density (mean IOD) = IOD/area.

### Transmission electron microscopy

RV tissue was dissected into 1 mm^3^ cubes and then treated with 2% glutaraldehyde in phosphate-buffered saline overnight at 4 °C, followed by post-fixation in 1% buffered osmium tetroxide. Sections of RV tissue were dehydrated in 70% acetone, and subjected to 1% phosphotungstic acid and 1% uranyl acetate solutions, followed by further dehydration in acetone. RV tissue sections were then embedded in resin and polymerized. Embedded tissues were then subject to ultrathin sectioning (60–70 nm) and contrast-staining with uranyl acetate and lead citrate, followed by imaging using a transmission electron microscopy (H-7650; Hitachi, Tokyo, Japan).

### Western blot analysis

Myocardial tissues were homogenized in phosphate buffer (150 mM NaCl, 50 mM Tris-HCl, pH 7.4, 1 mM EDTA, 1% Triton X-100, 1% sodium deoxycholate, and 0.1% SDS) with protease inhibitors and centrifuged at 12,000 rpm at 4 °C for 20 min to obtain supernatant. Equal amounts of protein were separated by 10% SDS-polyacrylamide gel and transferred onto nitrocellulose membranes. Membrane were incubated at 4 °C overnight with various primary antibodies, which included: rabbit anti-LC3 monoclonal antibody (1:1000, Cell Signaling Technology, Danvers, MA, USA); rabbit anti-p62 polyclonal antibody (1:1000, Cell Signaling Technology, Danvers, MA, USA); rabbit anti-mTOR and phospho-mTOR (Ser 2481) polyclonal antibodies (1:1000, Cell Signaling Technology, Danvers, MA, USA); rabbit anti-AMPKα and phospho-AMPKα ﻿(Thr172)﻿ monoclonal antibodies (1:1000, Cell Signaling Technology, Danvers, MA, USA); rabbit anti-p70S6 kinase and phospho-p70S6 kinase ﻿(Ser424) monoclonal antibodies (1:1000, Cell Signaling Technology, Danvers, MA, USA); rabbit anti-Beclin-1 monoclonal antibody (1:1000, Cell Signaling Technology, Danvers, MA, USA); mouse anti-bcl-2 monoclonal antibody (1:1000, Santa Cruz, CA, USA); rabbit anti-BNIP3 polyclonal antibody (1:1000, Cell Signaling Technology, Danvers, MA, USA); rabbit anti-HIF-1α polyclonal antibody (1:1000; Abcam, Cambridge, UK); and rabbit anti β-actin polyclonal antibody (1:500, Santa Cruz, CA, USA). Membranes were washed and subsequently incubated with HRP conjugated goat anti-rabbit secondary antibody (1:5000; ZSGB-Bio, Beijing, China) for 1 h at room temperature. Membranes were developed with enhanced chemiluminescence solutions, and protein bands were imaged using a UVP gel imaging system (UVP, Upland, CA, USA). Protein bands were digitized and subjected to densitometry with Image J software (National Institutes of Health, Bethesda, MD, USA). β-actin was used as a loading control to normalize the density of protein bands.

### Statistical analysis

All data were expressed as mean ± standard deviation. Data were statistically analyzed using Statistical Package for the Social Sciences v17.0 software (SPSS Inc., Chicago, IL, USA). An unpaired *t*-test was used to compare data between control and MCT rats at individual time points. One-way ANOVA then *q*-test were used for comparison of data from MCT rats at different time points. Correlations were determined by Spearman’s rank correlation coefficients. *P* < 0.05 was considered statistically significant.

## Results

### General features of MCT model

Previous studies have established the natural history of progression of RV hypertrophy to heart failure in the MCT model [2, 7]. MCT rats enter a phase of decompensation and heart failure in the fifth to sixth week after MCT injection mimicking signs of human PH associated with WHO class III and IV [2, 7]. Signs associated with RV failure included decreased physical activity, ascites, overall weight loss despite fluid retention (>20%), and increased mortality [2]. To mimic the natural history of RV remodeling progression associated with MCT in our study, we divided the animals into 2-, 4-, and 6- week subgroups. MCT treatment over 2 (MCT-2 W), 4 (MCT-4 W), and 6 (MCT-6 W) weeks caused death in 0% (0/12), 16.7% (2/12), and 33.3% (4/12) rats, respectively. In contrast, no rats died in the age-matched control subgroups during this induction period. In line with previous studies, [2, 7] we demonstrated that rats subjected to systemic MCT administration developed signs of PH (lethargy, respiratory distress, tachypnea) during the second week and gradually worsened during the fourth week. Subsequently, MCT rats presented typical RV failure symptoms during the sixth week, as described above. Of the 8 living rats in the MCT-6 W group, 6 of 8 presented with ascites and/or pleurisy, while all the rats showed decreased appetite and activity as well as obvious weight loss (Table 1). In clinical settings, mean pulmonary arterial pressure (mPAP) ≥25 mmHg at rest in patients, as assessed by right heart catheterization, is considered PH [36]. While previous study showed that a pulmonary artery systolic pressure (PASP) of 25 mmHg is the cut-off value for PH in the MCT rat model [37]. Altogether, we had 12, 10, and 8 living MCT rats, in the MCT-2 W, MCT-4 W, and MCT-6 W groups, respectively, included in our study.

**Table 1:** Cardiac hemodynamic and echocardiographic analyses of control and MCT-treated (over 2, 4, and 6 weeks) rats

Group	CON-2 W (*n* = 8)	MCT-2 W (*n* = 12)	CON-4 W (*n* = 8)	MCT-4 W (*n* = 10)	CON-6 W (*n* = 8)	MCT-6 W (*n* = 8)
RVSP (mmHg)	23.3 ± 1.8	30.1 ± 1.5^*^	23.9 ± 1.6	37.1 ± 2.7^**※^	24.7 ± 2.1	51.1 ± 3.7 ^**※※△^
RVWT (mm)	0.911 ± 0.09	0.976 ± 0.15^*^	0.924 ± 0.11	1.18 ± 0.16^**※^	0.932 ± 0.12	1.45 ± 0.13^**※※△^
RVEDD (mm)	36.3 ± 1.2	36.9 ± 1.1	37.9 ± 1.4	51.4 ± 2.9^*※※^	38.7 ± 1.9	58.9 ± 2.7^**※※△^
BW (g)	355 ± 13	344 ± 16	398 ± 14	361 ± 21^*※^	469 ± 17	357± 27^**※※△^
RVEDD/BW (mm/kg)	11.8 ± 0.8	10.7 ± 0.9	11.1 ± 0.7	14.1 ± 1.0^*※^	10.9 ± 0.9	17.3 ± 1.3^**※※△^
HR (bpm)	375 ± 9	379 ± 8	383 ± 10	403 ± 8	374 ± 10	419 ± 13^*^
TAPSE (mm)	0.28 ± 0.013	0.29 ± 0.019	0.29 ± 0.014	0.24 ± 0.015^*※^	0.30 ± 0.021	0.15 ± 0.026^**※※△^
RVEF (%)	61.5 ± 3.1	59.01 ± 2.1	63.8 ± 2.1	49.5 ± 3.6^*※^	62.7 ± 2.9	35.5 ± 6.0^**※※△^
RV FAC (%)	55.1 ± 2.9%	54.1 ± 3.3%	54.1 ± 3.1%	43.5 ± 5.1%^*※^	56.1 ± 2.7%	27 ± 3.9%^**※※△^
PAAT (ms)	32.2 ± 0.10	29.2 ± 0.15^*^	32.1 ± 0.11	25.9 ± 0.17^**※﻿﻿^	31.4 ± 0.10	22.6 ± 0.19^**※﻿※△^

*RVSP* Right ventricular systolic pressure, *RVWT* Right ventricular wall thickness, *RVEDD* Right ventricular diastolic diameter, *BW* Body weight, *HR* Heart rate (beats per minute), *TAPSE* Tricuspid annular plane systolic excursion, *RVEF* Right ventricular ejection fraction, *RV FAC* Right ventricular fractional area change, *PAAT* pulmonary artery acceleration time

Values are presented as mean ± standard deviation. ^*^ *P* < 0.05 and ^**^ *P* < 0.01, versus age-matched rat; ^※^ *P* < 0.05, ^※※^ *P* < 0.01, versus MCT-2 W; ^△^ *P* < 0.05, ^△△^ *P* < 0.01, versus MCT-4 W

### Hemodynamics and echocardiographic properties

**Fig. 1:**
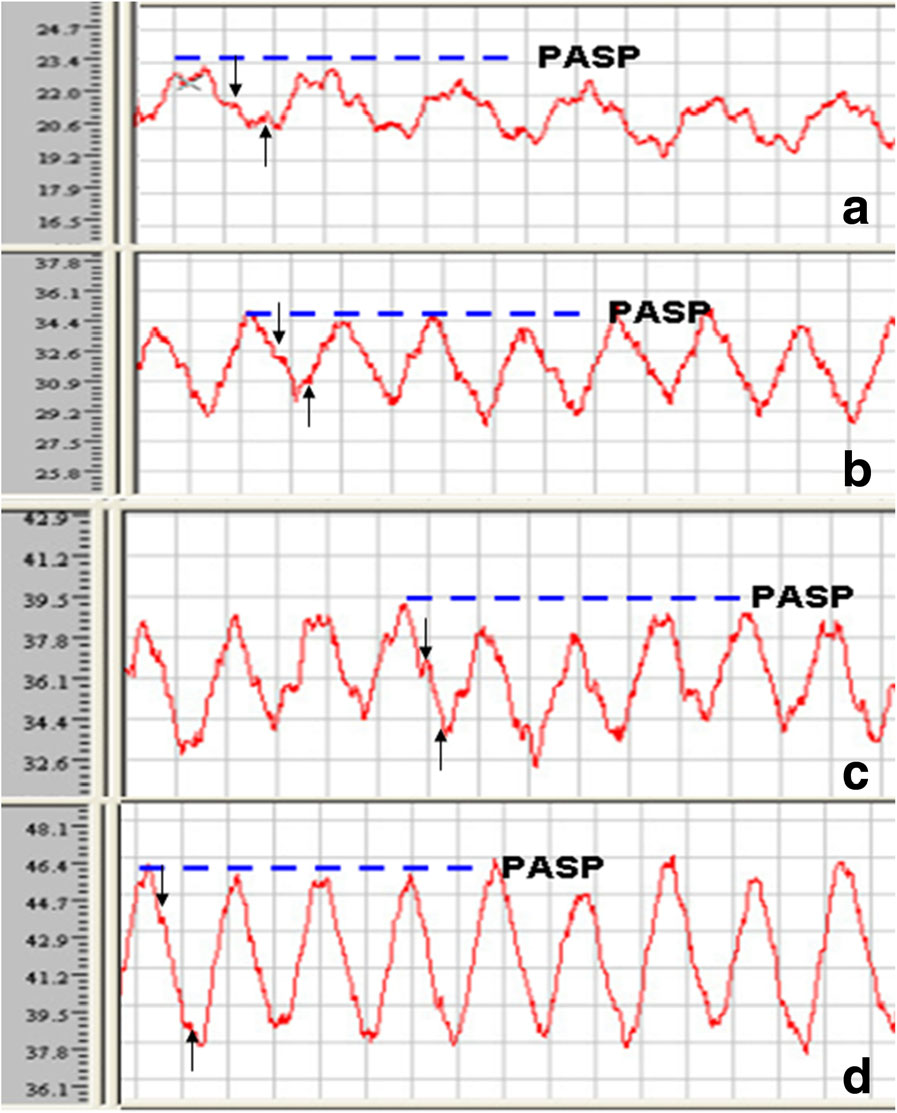
Representative pulmonary artery pressure waveforms obtained from control and MCT-treated rats. **a** Waveform image from control. **b**-**d** Waveform images from MCT-treated rats at 2 (MCT-2 W), 4 (MCT-4 W), and 6 (MCT-6 W) weeks, respectively. *Black downward arrowhead* points to the dicrotic notch, and *upward arrows* mark the diatolic recoil phase in the waveform images. Note: PASP, pulmonary artery systolic pressure

**Fig. 2:**
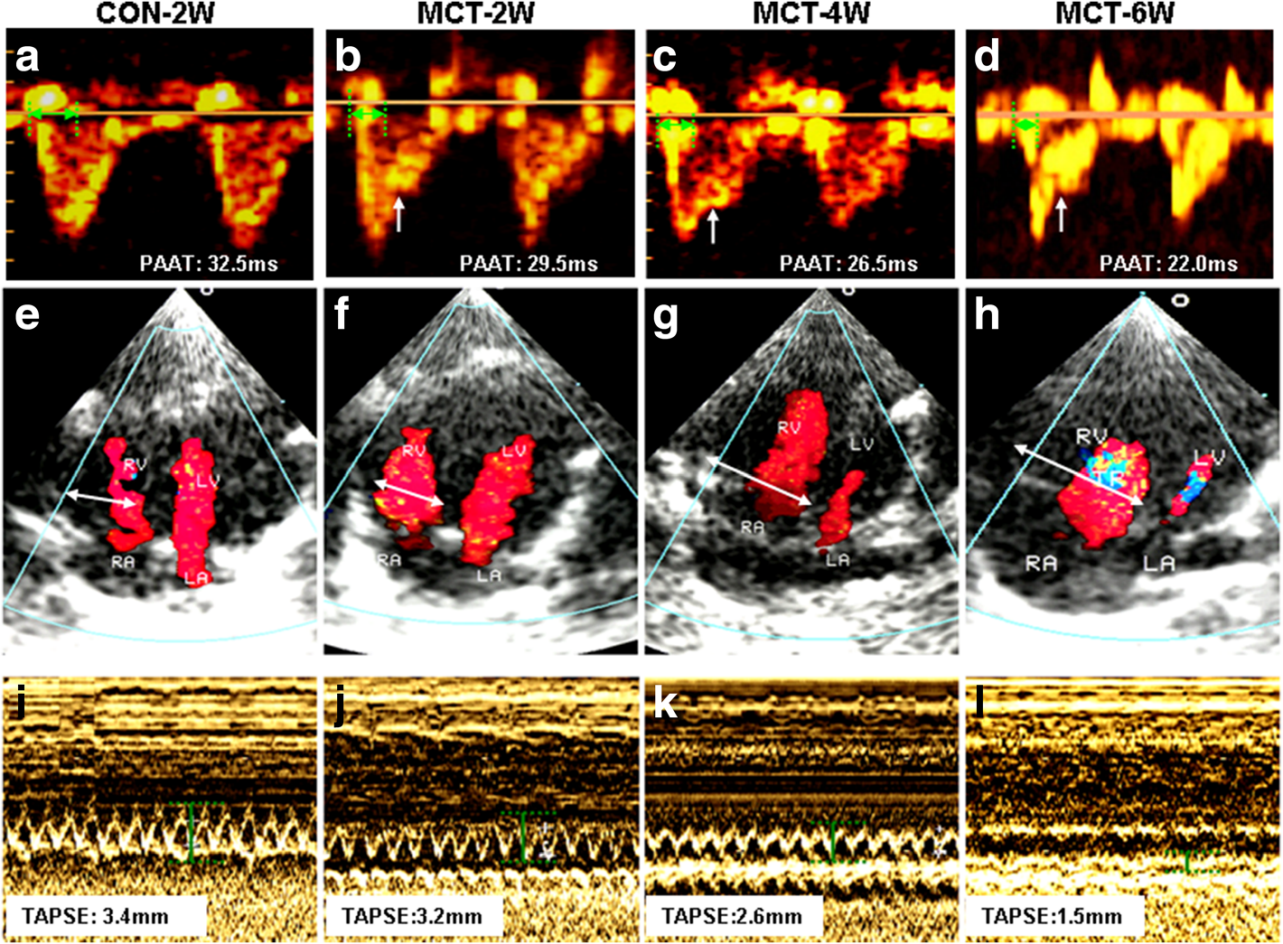
Representative echocardiography parameters obtained from control and MCT-treated rats at 2, 4, and 6 weeks. **a**-**d** Pulse-wave Doppler of parasternal view at the level of the pulmonary valve leaflets, which was aligned to maximize laminar flow. *White arrowhead* points to midsystolic pulmonary artery notching. Pulmonary artery acceleration time (PAAT) is highlighted by *green double-headed arrows*. **e**-**h** Morphology of right ventricle presented in four-chamber view. *White double﻿-﻿headed ﻿arrows * highlights RV internal diameter during diastole. **i**-**l** Tricuspid annular plane systolic excursion (TAPSE) highlighted by M-mode echocardiography

To validate the PH and RV overload in our MCT model, right heart catheterization was performed. The PASP in MCT groups was significantly elevated compared to age-matched controls (*P* < 0.05). Moreover, PASP progressively increased with extended treatment times in MCT rats (Table 1, Fig. 1). Pulmonary artery waveforms highlight the presence of a dicrotic notch and a diastolic recoil phase after the dicrotic notch, which is widely regarded as a sign showing the right heart catheter has advanced into the pulmonary artery (Fig. 1) [30, 31]. The high pulmonary arterial pressure in the MCT groups was also reflected by the shorter pulmonary artery acceleration time (Table 1, Fig. 2a-d) and the appearance of systolic notching in the Doppler flow waves, compared with the normal-looking parabolic waves found in control rats. To further verify whether MCT could recapitulate the progressive RV remodeling in PH, echocardiography was carried out to directly assess RV dimensions and systolic function. It has been established that TASPE of <17.5 mm in the MCT model, [38] RVEF of <40% [39] and RV FAC of <35% [28] in clinical settings are widely used as gauges for RV systolic dysfunction. TAPSE, RVEF, and RV FAC were decreased, while RV end-diastolic dimension (RVEDD) increased in MCT-4 W and MCT-6 W subgroups compared to the controls (Table 1, Fig. 2e-i). TAPSE, RVEF, and RV FAC reached above cut-off values, which was consistent with RV failure symptoms in the MCT-6 W subgroup.

### RV myocardial histology process

**Fig. 3:**
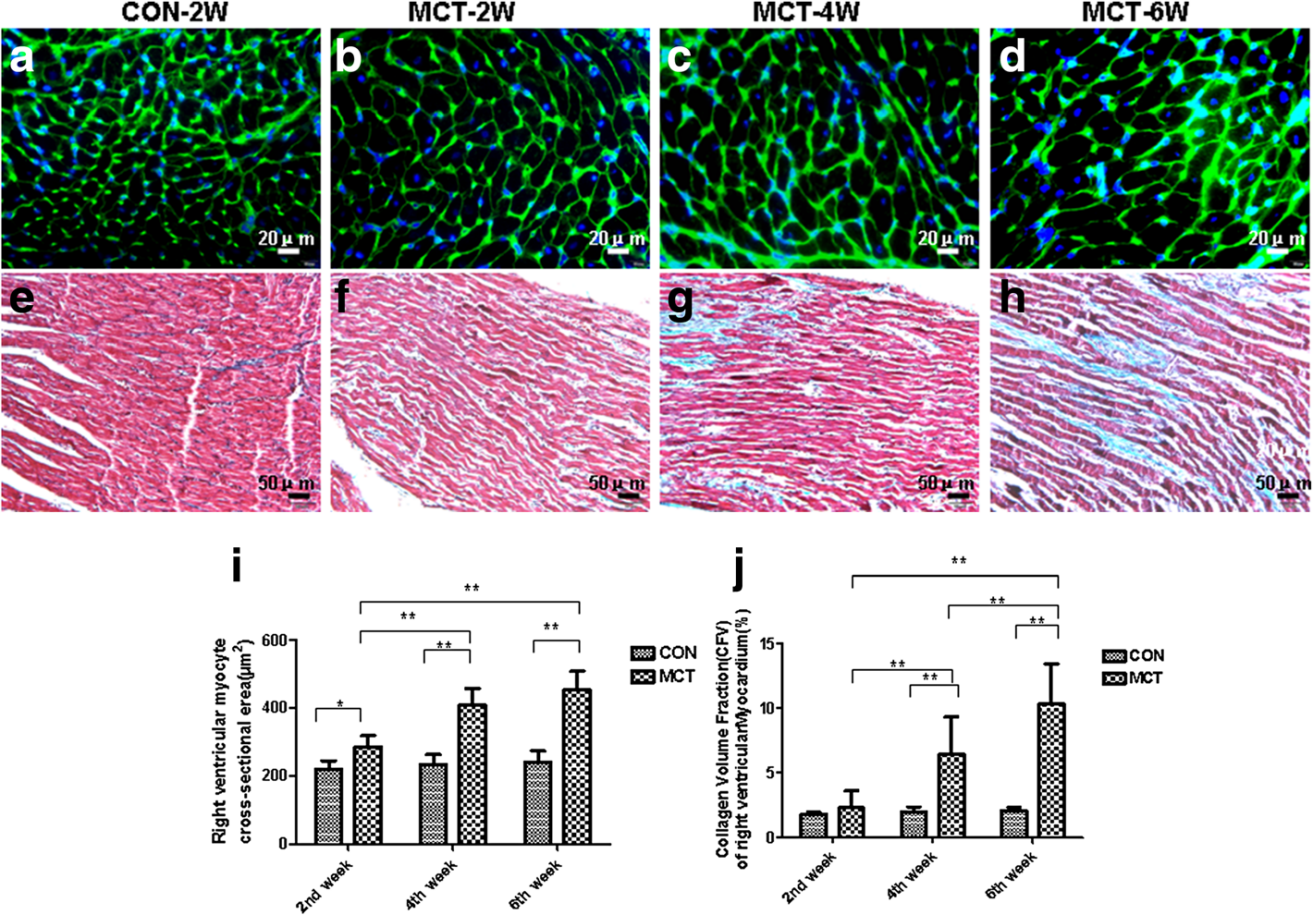
Histological alterations in the RV during compensatory hypertrophy and heart failure in the MCT-treated rats. **a**-**d** Cardiomyocyte cross-sectional area and myocardial interstitial fibrosis [using wheat-germ-agglutinin staining (WGA) and Masson trichrome staining, respectively] from control and MCT rat model. Representative images of WGA stained cardiomyocytes from control and MCT treated rats at 2, 4, and 6 weeks. *White* scale represents 20 μm (×400 original magnification); (**e**-**h**) Representative images of Masson trichrome-stained RV tissues from control and MCT treated rats at 2, 4, and 6 weeks. *Red* stain denotes muscle fibers. *Blue* stain denotes collagen. *Black* scale represents 50 μm (×200 original magnification); (**i**) Quantitative analysis of cardiomyocyte cross-sectional (transverse) area; (**j**) Quantitative analysis of interstitial fibrotic area. ^*^ *P* < 0.05, ^**^ *P* < 0.01. Values are presented as mean ± standard deviation

To corroborate the echocardiography findings, RV tissues were assessed by quantitative histological analysis. Consistent with previous findings [2, 7], MCT-treated rats showed a gradual increase in RV/BW and RV/(LV + IVS) ratio from the second to sixth weeks (Table 2). Similarly, RV cardiomyocyte cross-sectional area progressively increased from the second to sixth week after MCT treatment, as observed by WGA stain (Fig. 3a-d, i). Myocardial fibrosis calculated by CVF was also increased in RV tissues from MCT-4 W and MCT-6 W subgroups compared to the MCT-2 W subgroup and to a greater extent compared to age-matched controls (Table 2, Fig. 3e-h, j).

**Table 2:** Cardiac morphometry parameters in control and MCT-treated (at 2, 4, and 6 weeks) rats

Group	CON-2 W (*n* = 8)	MCT-2 W (*n* = 12)	CON-4 W (*n* = 8)	MCT-4 W (*n* = 10)	CON-6 W (*n* = 8)	MCT-6 W (*n* = 8)
RV/LV + IVS (g/g)	0.24 ± 0.02	0.27 ± 0.02^*^	0.25 ± 0.02	0.48 ± 0.06^**※※^	0.23 ± 0.03	0.62 ± 0.09 ^**※※△^
RV/BW (g/kg)	0.48 ± 0.02	0.53 ± 0.02^*^	0.49 ± 0.04	0.85 ± 0.06^**※※^	0.45 ± 0.03	1.22 ± 0.07^**※※△^

*BW* Body weight, *LW* Lung weight, *RV* Right ventricle, *LV* Left ventricle, *IVS* Interventricular septum, *MCD* Myocardium cell diameter

Data are presented as mean ± standard deviation. ^*^ *P* < 0.05 and ^**^ *P* < 0.01, versus age-matched rat; ^※^ *P* < 0.05, ^※※^ *P* < 0.01, versus MCT-2 W; ^△^ *P* < 0.05, versus MCT-4 W

### Autophagy signature in the RV of MCT rats

**Fig. 4:**
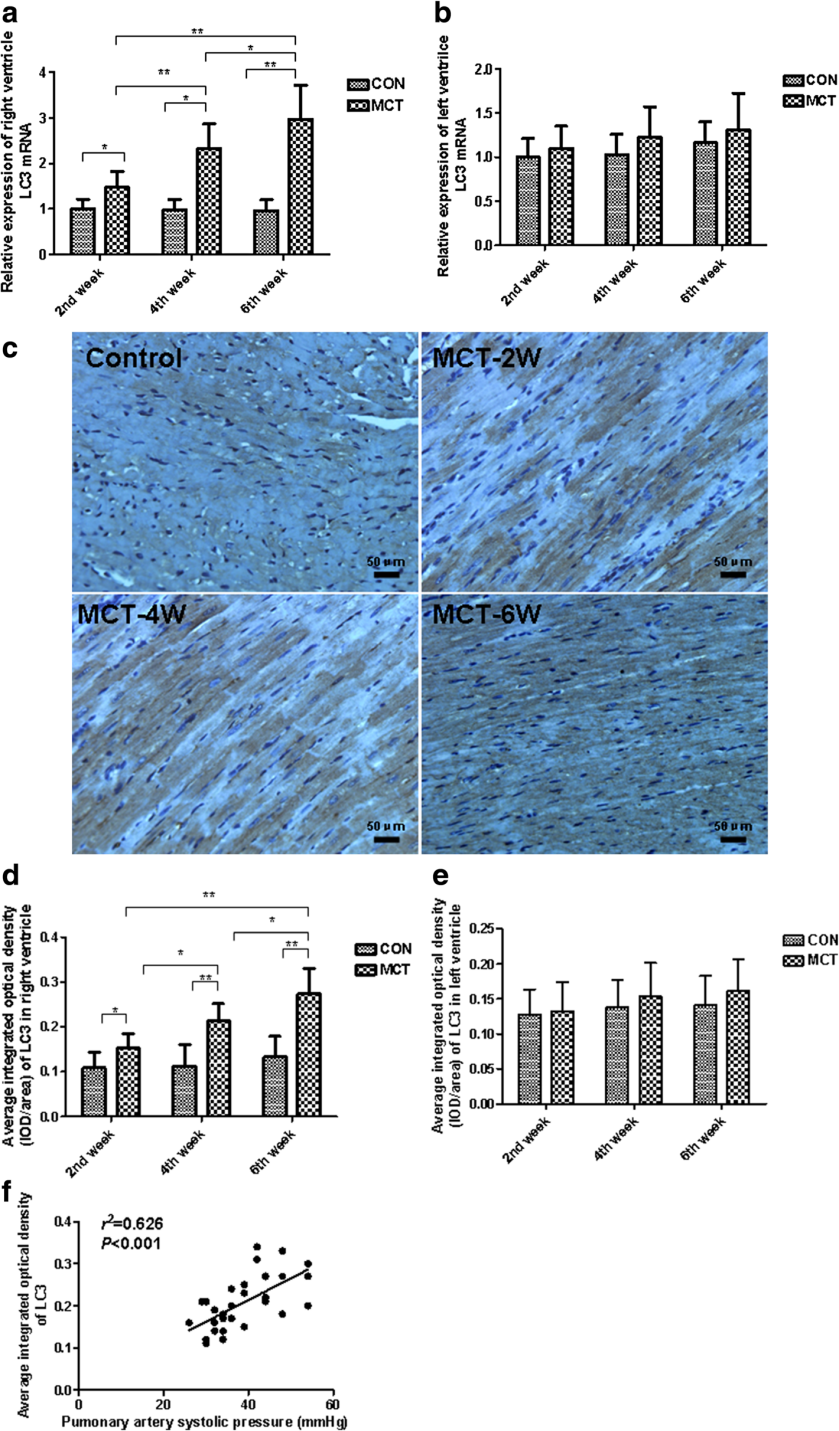
Expression of the autophagic marker, LC3, is upregulated in the RV of the MCT rat model. **a**, **b** RT-*q*PCR of LC3 mRNA expression relative to GAPDH in RV (*left*) and LV (*right*) tissue of control and MCT-treated rats. GAPDH was used as a loading control. **c**, **d** Representative RV tissue sections immunostained for LC3 protein (*dark brown*) in control and MCT-treated rats. Note: *Dark brown* granules in myocardial cell cytoplasm. *Black* scale represents 50 μm (×200 original magnification). **e**, **f** Quantification of LC3 positive protein signal (integrated optical density) in RV (*left*) and LV (*right*) tissue sections per field area. **g** Correlation of pulmonary artery systolic pressure with LC3 positive protein signal in 2, 4, and 6 week MCT-treated rats. ^*^ *P* < 0.05, ^**^ *P* < 0.01. Values are presented as mean ± standard deviation

The main objective of the study was to examine the time course of changes in autophagy in the RV subsequent MCT injection. Using RT-*q*PCR and immunochemistry, we assessed the expression of the autophagosomal marker LC3, which is a key protein involved in the regulation of autophagy. We found that LC3 mRNA and LC3 protein (as measured by densitometry) became markedly and gradually upregulated in the RV myocardium during the progression of PH in MCT rats, and reached its peak at sixth week after MCT treatment (Fig. 4a-c). In contrast, both LC3 mRNA and LC3 protein remained low and constant in the controls at every time point assessed after MCT treatment. Additionally, no significant differences in these parameters could be observed in the LV of MCT rats at these different time points. Interestingly, LC3 levels in the MCT-treated groups strongly correlated to pulmonary artery systolic pressure (PASP), (*r* ^2^ = 0.626, *P* < 0.01) (Fig. 4d).

**Fig. 5:**
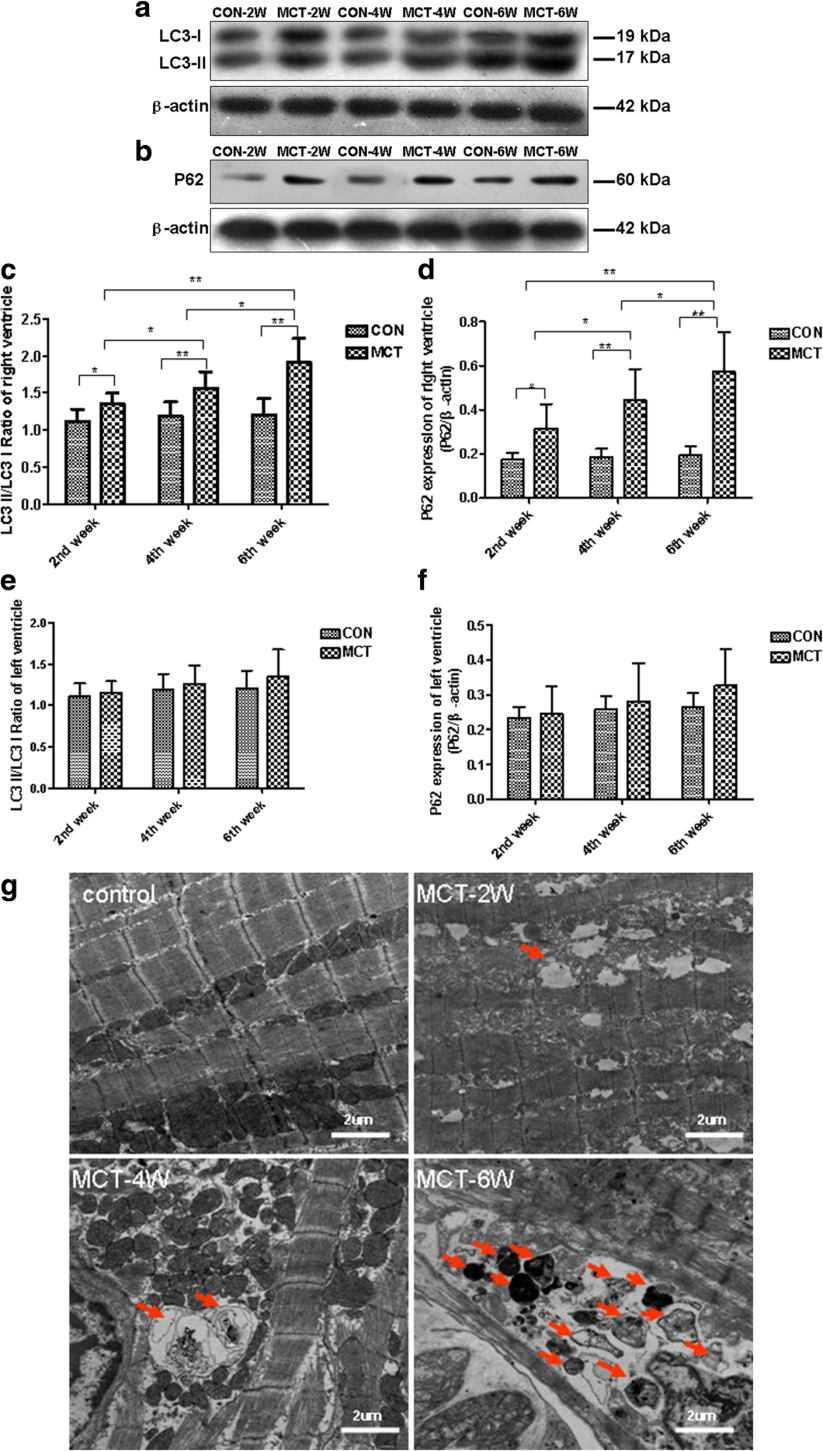
Autophagic induction in the RV of MCT rats. **a**, **b** Protein blot analysis of autophagy-related proteins, LC3II, LC3I, and p62 in RV protein extracts from control and MCT-treated rats. β-actin was used as a loading control. **c**-**f** Quantification of LC3II to LC3-I ratio (*right*) and p62 (*left*) protein expression normalized to β-actin levels. **g** Representative transmission electron micrographs from RV of control and MCT-treated rats. *Arrows* indicate autophagosomes. *White* scale represents 2 μm (15,000× original magnification). Values are presented as mean ± standard deviation. ^*^ *P* < 0.05, ^**^ *P* < 0.01

Western blot analysis was further used to quantify LC3-II/LC3-I ratio and p62 expression in rats. We showed that the RV of MCT rats exhibited significantly higher LC3-II/LC3-I ratios and p62 expression when compared to age-matched controls, which reached peak during the sixth week of MCT treatment (Fig. 5a and b). Also, no significant difference in levels of these proteins could be observed in the LV of MCT-treated rats (Fig. 5c).

Transmission electron microscopy (TEM) was utilized to verify that autophagosomes and morphological evidence of autophagy activation were present in RV tissue of MCT-treated animals. Characteristic ultrastructural features of autophagosomes include a single or double membraned structure that encompasses organelle remnants and cellular cargo (arrow in Fig. 5d). Their presence gradually intensified during the RV disease progression in the MCT model. Thus, autophagosomes were the most abundant within RV cardiomyocytes of MCT-treated rats in the 6-week group. In contrast, autophagosomes were rarely observed and autolysosomes were never observed in controls Fig. 5d).

### Distinct activation of pro-autophagic signaling pathways

**Fig. 6:**
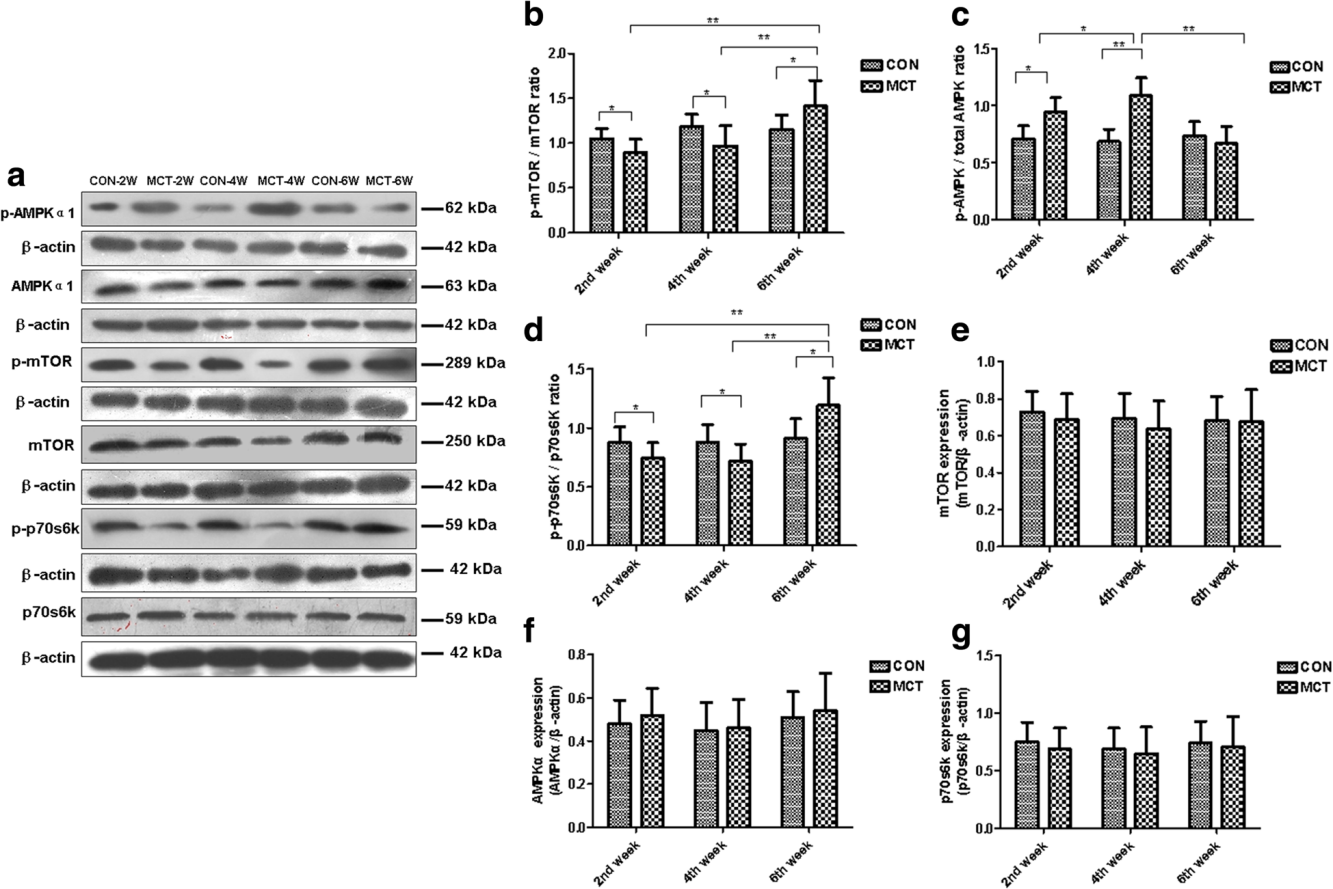
AMPK-dependent mTOR signaling pathways are distinctly dysregulated in RV of MCT rat model. **a** Western blot analysis of total and phosphorylated levels of AMPK, mTOR, and p70S6 kinase in RV protein extracts from control and MCT treated rats for 2, 4, and 6 weeks. β-actin was used as a loading control. **b**-**d** Quantification of phosphorylated levels of AMPK, mTOR, and p70S6K to total AMPK, mTOR and p70S6K ratios. **e**-**g** Quantification of AMPK, mTOR, and p70S6 protein band signal densities was performed using ImageJ and plotted normalized to β-actin levels. Values are presented as mean ± standard deviation. ^*^ *P* < 0.05, ^**^ *P* < 0.01

Another objective of the study was to determine the signaling pathways that may be involved in RV cardiac autophagy in the MCT model, including mTOR (Ser 2481), AMPKα (Thr172), p70S6K (Ser424), Beclin-1 HIF-1α, Bcl2, and BNIP3. Using Western blot analysis, we assessed the protein expression of each signaling component in MCT-treated rats and controls at each time point. Compared to controls, downregulation of phosphorylated mTOR (normalized to total level) was observed in RV of the MCT-2 W and MCT-4 W rats; phosphorylated mTOR became upregulated in the RV of the MCT-6 W group rats (Fig. 6a and b) This regulation is likely achieved through a AMPK mechanism, as we observed phosphorylated AMPKα (normalized to total level) trended towards increased activation in the RV of the MCT-2 W and MCT-4 W rats compared to controls; however, it appears to decline in the RV of MCT-6 W rats compared with the MCT-2 W and MCT-4 W groups (Fig. 6a and c). Similarly, phosphorylated p70S6K expression (normalized to total level), which is downstream of mTOR, paralleled the changes observed for phosphorylated mTOR (Fig. 6a and d). No changes in total mTOR, AMPKα, and p70S6K were observed in the different groups (Fig. 6a and e–h).

**Fig. 7:**
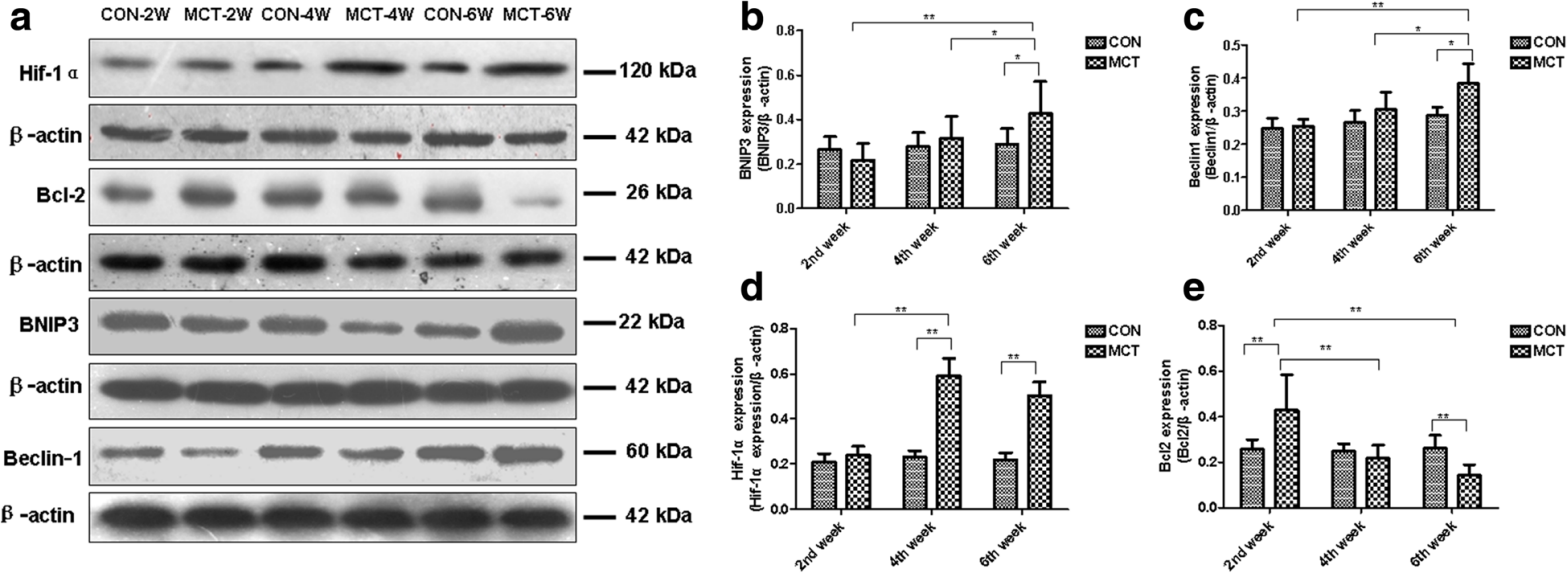
HIF-1α/Bcl2/BNIP3/Beclin-1 signaling pathways are distinctly dysregulated in RV of MCT rat model. **a** Western blot analysis of HIF-1α, Bcl2, BNIP3 and Beclin-1 protein in RV protein extracts from control and MCT-treated rats for 2, 4, and 6 weeks. β-actin was used as a loading control. **b**-**e** Quantification of HIF-1α, Bcl2, BNIP3, and Beclin-1 protein band signal densities was performed using ImageJ software and plotted normalized to β-actin levels. Values are presented as mean ± standard deviation. ^*^ *P* < 0.05, ^**^ *P* < 0.01

In contrast, expression of BNIP3 and Beclin-1 was relatively low in the RV of the MCT-2 W and MCT-4 W groups, and then became significantly upregulated in the MCT-6 W group compared with age-matched controls (Fig. 7a–c). However, it appears that the BNIP3 activation was not totally dependent on Hif-1α. HIF-1α levels were slightly increased in the RV of the MCT-2 W group and became significantly increased in the RV of the MCT-4 W group, but then slightly decreased in MCT-6 W group (Fig. 7a, d). Additionally, Bcl2 peaked in expression in the RV of the MCT-2 W group, and then went on a gradual decline in the MCT-4 W and MCT-6 W groups. Again, no significant differences could be observed in autophagy signaling in controls at these different time points (Fig. 7a, f).

## Discussion

To the best of our knowledge, this is the first study to investigate the time course of autophagy and pro-autophagy signalings in the RV myocardium of the MCT model. The key findings of the present study suggest that sustained activation of autophagy is associated with increased pulmonary artery systolic pressure. Moreover, AMPK mediated mTOR as well as BNIP3-dependent Beclin-1 autophagic signaling pathways were involved at distinct stages during RV remodeling of the MCT model.

### Modeling the clinical stages of progression of RV hypertrophy to failure in the MCT rat

Studies focused on RV remodeling in PH present a single “snapshot in time” without separating between the stages of compensatory hypertrophy and RV failure. In addition, animal models (i.e., PAB model, MCT model, and Sugen/hypoxia models) are used to duplicate aspects of compensatory hypertrophy or overt decompensated states of the RV in order to investigate common mechanisms underlying RV remodeling, respectively. This may be problematic as the cardiac hemodynamics and mechanisms of RV remodeling in different animal models may differ in terms of mechanisms and severity [34, 40]. To avoid potential confounding effects, Paulin and Sutendra objectively measured hemodynamic criteria, clinical features, and echocardiography of the MCT rat model to establish the time course of the specific RV remodeling stages [2, 7].

Considering that autophagy may play a different role during different phases, [20] we sought to determine the autophagy signatures during the switch from compensatory hypertrophy to failing in the RV within the MCT model, as it progresses to PH. To reduce the variation of rats included, the rats at each time point were also carefully phenotyped (e.g., with hemodynamic, clinical, and echocardiography characteristics). Consistent with previous findings [2, 7], after 2 weeks of MCT treatment, the rats experience PH and a slight increase in RV myocardial hypertrophy in the absence of changes in dilatation, which attests to the fact that the RV is likely in the early compensatory phase. At 3–4 weeks after MCT treatment, rats manifest RV enlargement, while RV function is moderately decreased (but do not reach RV failure cut-off values) indicative of compensatory hypertrophy [2, 7]. At 5–6 weeks after MCT treatment, rats exhibited typical characteristics of RV decompensation and failure (sharp decrease in RV function parameters which reach RV failure cut-off values for TAPSE, RV FAC, and RVEF by echocardiography) resulting in significant mortality. These data highlight how the MCT rat model is characterized by RV failure in vivo.

### Dynamic changes of autophagy in the progression RV hypertrophy toward heart failure

A novel aspect of our data is the discovery of the time course of autophagy-related changes from compensatory hypertrophy to failure stages in the RV. Our results show sustained activation of autophagy in the RV of the MCT model—as evidenced by the presence and increased expression of the autophagy marker LC3 as well as LC3-II/LC3-I ratio. This is consistent with Qipshidze’s finding showing a marked increase in LC3 protein expression in the RV of a PAB model [16]. In our work, the upregulation of autophagy was further evidenced by the gradual increase in the clustered accumulation of autophagosomal structures in RV observed using a transmission electron microscope. Considering the dynamic nature of autophagy, the abundance of autophagosomes can reflect either induction of autophagosome formation or a defect of autophagosome degradation. P62 is an adaptor protein that binds to ubiquinated protein products destined for consumption by the autophagosome machinery. As such, accumulation of p62 can be used as a marker of proteotoxicity build-up within the cell and a decrease in the function of autophagic clearance mechanisms [14, 15]. We demonstrated the sustained upregulation of p62 during the progression of RV remodeling, suggesting that the autophagosome clusters may be due to an increase in autophagy activation, rather than inhibited autophagosome degradation. It has been shown that the MCT model does induce a liver toxicity response; however, whether it affects autophagy in the RV remains unclear. Interestingly, our data revealed no obvious changes in autophagy in the LV at different stages of the MCT model, thus highlighting the chamber-specific nature of the autophagy induction to the RV owing to the afterload-related changes associated with PH, as opposed to the MCT-induced response.

Cardiac hypertrophy is an important feature of cardiac remodeling and a recent study suggested the importance of autophagy in the pathogenesis of cardiac hypertrophy as it regulates cardiomyocyte protein degradation. Nakai et al. found that knockdown of the autophagy-related protein, autophagy-related gene 7 (Atg7), using RNAi inhibited autophagy in rat neonatal cardiomyocytes, which was sufficient to induce cardiomyocyte hypertrophy [9]. In vivo studies by the same group showed that rapid ablation of autophagy-related gene 5 (Atg 5) by tamoxifen treatment of an inducible cardiac-specific Atg5-deficient mouse model resulted in an increase in cardiomyocyte cross-sectional area [9]. More recent reports have revealed that inhibition of mTOR-mediated protein synthesis was critical for preventing pathological hypertrophy by regulating autophagy [23, 41]. In the present study, we showed that phosphorylation of mTOR in the RV was significantly suppressed in the 2- and 4- week MCT model, coinciding with an increase in the autophagy marker LC3-II/LC3-I ratio, suggesting that activation of autophagy at this stage may be an important target to potentially circumvent RV myocardial hypertrophy. Additionally, we found that the upregulation of LC3 levels in the RV correlated with increased PASP. This finding supports the hypothesis that increased RV pressure afterload activate oxidative stress—caused by excess reactive oxygen species (ROS) excess, which can play a vital role in activating autophagy [16, 42]. Indeed, a previous study has shown that ROS can inhibit autophagy-related gene 4 (Atg4), activity through oxidation of an essential cysteine residue, which can then block the cleavage of phosphatidylethanolamine (PE) from PE-conjugated LC3 and promote autophagy [43].

### Alterations and potential role of autophagy signaling pathways

Ample evidence suggests autophagy has a principal role in pro-survival and pro-death mechanisms dependent on the activation of different signaling pathways [8, 44]. Thus, the potential pathways involved in autophagy regulation were studied. It is noteworthy that both the inhibition of AMPK/mTOR or the activation of HIF-1α/Bcl2/ BNIP3/Beclin-1 pathways can upregulate autophagy [22]. The most intriguing finding from our study is that although persistent activation of autophagy was observed during RV failure progression, the underlying autophagic signaling patterns varied. AMPK is a protein kinase activated in response to low/depleting ATP levels. AMPK promotes cytoplasmic adenosine monophosphate (AMP) to serve as a master negative regulator of autophagy by phosphorylating and activating proteins of the tuberous sclerosis 1/2 complex, which then act to deactivate phospho-mTOR and phospho-70s6K [45, 46]. We highlight the potential reliance of autophagic activation via AMPK-dependent inhibition of mTOR during the compensatory phase (2 and 4 weeks after MCT treatment). This observation fits with the current understanding that mild ischemic stress can activate AMPK-mediated inhibition of mTOR-dependent autophagy pathways [46]. Although it remains unclear why AMPK activity significantly declines during end-stage heart failure, a similar change has been noted recently in rat cardiomyocytes with ischemic conditioning [23]. Interestingly, upregulation of AMPK has also been observed in the LV in hypertension; however, no inhibition of mTOR and p70s6K was observed in the LV of the hypertensive rat [13]. This discrepancy may be attributed to the chamber-specific stress response to overload and differences in disease models. A previous study on autophagy induction during ischemia and glucose deprivation demonstrated that AMPK activation accompanied mTOR inactivation in cardiomyocytes [46]. Inhibition of AMPK was shown to cause a significant reduction in autophagy, while cardiomyocyte survival was decreased by pharmacological inhibition of AMPK, suggesting a protective role for AMPK-dependent autophagy in cardiomyocytes [46]. These findings altogether suggest a protective role for autophagy in cardiac hypertrophy during the compensatory phase.

Another interesting finding from our study involves autophagic activation via BNIP3 and Beclin-1 overexpression during RV failure (6 weeks after MCT treatment). BNIP3 is an important regulator of cardiomyocyte mitochondrial function and survival during ischemic/hypoxia injury [10, 47]. That is, the mitochondrial perturbations triggered by BNIP3 gene activation were found to parallel opening of the mitochondrial permeability transition pore, mitochondrial membrane potential loss, and cell death [48]. Although HIF-1α is an upstream molecular target of BNIP3, [22] we showed that HIF-1α did not completely parallel the alterations in BNIP3, suggesting that there may be other unrecognized upstream molecular pathways involved in BNIP3 regulation in RV failure. The role of autophagy at this stage is intriguing. A recent study demonstrated that BNIP3-deficient mice exhibited diminished cardiac dilatation and preserved ventricular systolic performance post-infarction [49]. In contrast, BNIP3 overexpression induced progressive ventricular dilation and impaired systolic performance possibly partly due to increased mitochondrial apoptosis and mitophagy [50]. Consistent with this previous study, [51] we provided evidence that BNIP3 may induce mitochondrial fragmentation and autophagy. Beclin-1 is a downstream target of BNIP3. Beclin-1 haploinsufficient mice exhibited decreased autophagic activity, preserved cardiac performance, and partial inhibition of cardiomyocyte death after aortic banding [9]. Conversely, cardiac-specific Beclin-1 overexpressing mice resulted in an amplified pathological remodeling response [52]. In addition to regulating Beclin-1, BNIP3 may also directly act on the important apoptosis regulator Bcl2 and trigger cell death [50]. This role may explain potential cross-talk between autophagy and apoptosis in RV remodeling in PH. This finding is supported the prevailing view that maladaptive autophagy could inducing cell death [9]. The data gained from our study has provided opportunities to hypothesize a cooperative role for autophagy and apoptosis in driving adverse pathological remodeling of the RV by BNIP3 and Beclin-1-dependent autophagy activation.

To date, no medications specifically target myocardial autophagy. However, based on our new findings that identify autophagy-associated targets, we believe that use of mTOR inhibitors (e.g., rapamycin), AMPK activators (e.g., AICAR), and development and use of selective BNIP3 inhibitors in the late stages of RV compensation/decompensation might be helpful to improve RV function and remodeling. Our findings also support previous studies that demonstrate that treatment with rapamycin, an inhibitor of mTOR, resulted in prevention of RV hypertrophy and dysfunction in an animal model of PH [53]. Thus, rapamycin may not only improve vascular remodeling in the lung, resulting in an improvement in RV-pulmonary artery coupling, but may also have direct effects on autophagy regulation and thus, protection of the RV.

### Study limitations

Our study has some limitations. Firstly, we did not investigate the effects of selectively upregulating and inhibiting autophagy by pharmaceutical agents at different stages of RV remodeling in PH. This was not performed in the current study owing to the present limitation in identifying pharmacological reagents that selectively target cardiac autophagy. Secondly, another potential limitation of our study is that the autophagy signatures in the RV of the MCT model associated with PH could be very different from other animal models of PH due to hypoxia, Sugen/ hypoxia, hyperflow, [34] and the PAB rat model [54]. For example, the PAB model impacts RV hypertrophy but has no impact on the pulmonary vasculature—possibly one of the important causes of RV hypoxia in the MCT model that may also impact autophagy in the RV [29]. Thus, whether our findings can apply to other models warrants further study.

It is important to also note that it remains unclear whether the findings in the MCT model can be generalized to humans. Thus, future studies should also consider the assessment of autophagy signatures in RV tissues, of PH patients with different etiologies. Furthermore, fibrosis is an important feature of RV remodeling; however, our study does not assess a causality chain between myocardial fibrosis and these autophagic signaling pathways. Thus, comprehensive in vivo and in vitro studies are required to test this hypothesis in the future.

## Conclusions

Our findings highlight that persistent up-regulated autophagy was associated with increased pressure overload. Most importantly, different signaling mechanisms (mTOR and Beclin-1 mediated pathways) are involved in autophagy activation of the RV in the MCT rat model. These findings may have broad implications in better designing pharmacological strategies to protect against RV remodeling and dysfunction in PH in a stage-specific manner.

## Abbreviations

AMP: Adenosine monophosphate
AMPKα: Adenosine monophosphate-activated protein kinase alpha subunit
BCL-2: B-cell lymphoma 2
BNIP3: Protein-interacting protein 3
BW: Body weight
CVF: Collegen volume fraction
HIF-1α: Hypoxia inducible factor 1α
IVS: Interventricular septum
LC3: Microtubule associated protein light chain 3
LW: Lung weight
MCD: Myocardium cell diameter
MCT: monocrotaline
mTOR: mammalian target of rapamycin
P62: Hypothetical protein
PAAT: Pulmonary artery acceleration time
PASP: Pulmonary artery systolic pressure
PE: Phosphatidylethanolamine
PH: Pulmonary hypertension
RA: Right atrium
ROS: Reactive oxygen species
RV FAC: Right ventricular fractional area change
RVEDD: Right ventricular end-diastolic diameter
RVEDV: Right ventricular end-systolic volume
RVEF: RV ejection fraction
RVESV: Right ventricular end-diastolic volume
RVF: Right ventricular failure
RVWT: Right ventricular wall thickness
SVC: Superior vena cava
TAPSE: Tricuspid annular plane systolic excursion
